# Effectiveness of a Systematic Epidemic Prevention Program on the Coping Response of Nursing Staff Caring for High‐Risk COVID‐19 Patients

**DOI:** 10.1002/nop2.70600

**Published:** 2026-06-04

**Authors:** Shui‐Tao Hu, Wen‐Pin Yu, Fang‐Kuei Hsieh, Chien‐Tzung Chen

**Affiliations:** ^1^ Department of Nursing Keelung Chang Gung Memorial Hospital Keelung City Taiwan; ^2^ Department of Nursing Chang Gung University of Science and Technology Taoyuan City Taiwan; ^3^ Department of Nursing Linkou Chang Gung Memorial Hospital Taoyuan City Taiwan; ^4^ Office of Superintendent Linkou Chang Gung Memorial Hospital Taoyuan City Taiwan; ^5^ College of Medicine Chang Gung University Taoyuan City Taiwan

**Keywords:** high‐risk COVID‐19, nursing staffs, systematic epidemic prevention programme

## Abstract

**Aims:**

To explore the effectiveness of a systematic epidemic prevention programme on the coping response of nursing staff caring for high‐risk COVID‐19 patients.

**Design:**

A one‐group pre‐post‐test pre‐experimental design was used.

**Methods:**

Through purposive sampling, a total of 84 nursing staff were recruited from a teaching hospital who had experiences in caring for high‐risk COVID‐19 patients. The participants underwent a systematic epidemic prevention programme. Before the intervention and 1 month, 3 months, and 6 months after the intervention at four time points, the coping response of the nursing staff was measured through three scales—the Impact of Event Scale‐Revised, the General Health Questionnaire (GHQ), and the Brief Coping Orientations to Problems Experienced (Brief‐COPE). The data were analyzed using descriptive and inferential statistics including generalised estimating equations, Pearson's correlation coefficient, independent samples *t*‐test, and analysis of variance.

**Results:**

The systematic epidemic prevention programme significantly improved nursing staff's coping responses. IES‐R scores decreased over time but did not reach statistical significance. GHQ scores showed a significant time effect, with reductions observed at 1 and 3 months post‐intervention, and the greatest improvement at 3 months. Emotional coping significantly increased at 3 months post‐intervention. Overall, the findings demonstrate a sustained improvement in coping responses across time points following the intervention.

**Reporting Method:**

The study followed the TREND and TIDieR checklists.

**Patient or Public Contribution:**

None.

## 1 Introduction

Coronavirus disease 2019 is a respiratory disease caused by a newly discovered coronavirus and is sometimes referred to as severe infectious pneumonia or coronavirus pneumonia. Individuals infected with COVID‐19 may experience a mild to severe respiratory illness with symptoms that include fever, weakness, dry cough, nasal congestion, headache, sore throat, hemoptysis, and diarrhoea, and potentially death. The number of confirmed cases worldwide has reached 228,628,675, including 4,707,500 deaths, for a fatality rate of 2.06%. The confirmed number of cases in Taiwan has reached 16,152, including 840 deaths, for a mortality rate of 5.20% (Taiwan Centers for Disease Control (CDC) 2021). The World Health Organization (WHO) warned that medical personnel at the forefront of the COVID‐19 response were at a high risk of infection due to pathogen exposure and were also subject to long working hours (WHO 2020), psychological distress, and physical and mental fatigue. When public health or medical emergency occurs, such as an infectious disease, the physical and mental reactions of the nursing staff who care for patients will be affected (Phua et al. 2005). The following systematic epidemic prevention programme was established to improve the coping response to COVID‐19.

## 2 Background

The COVID‐19 pandemic has placed substantial psychological and occupational burdens on nursing staff, particularly those caring for high‐risk patients. Nurses are at the frontline of care and are frequently exposed to infection risk, high workloads, and prolonged stress, which may lead to significant mental health challenges. Previous studies during infectious disease outbreaks, such as SARS and COVID‐19, have documented that healthcare workers commonly experience fear, anxiety, insomnia, and emotional distress (Maunder et al. 2003; Nickell et al. 2004; Reynolds et al. 2008). These psychological symptoms not only affect personal well‐being but also pose a threat to the quality of patient care.

Post‐traumatic stress disorder (PTSD) and coping responses are two critical constructs for understanding psychological adaptation in these high‐stress environments. PTSD reflects the psychological impact of exposure to traumatic events, while coping strategies represent the behavioural efforts individuals use to manage such stress. Recent global evidence highlights the variability of these responses; for instance, healthcare workers in Australia and Italy demonstrated a mix of approach and avoidance strategies, with cognitive restructuring and emotion‐focused strategies playing key roles in resilience (Fiorillo and Gorwood 2020; Smallwood et al. 2021). Effective coping has been associated with better mental health outcomes, whereas maladaptive strategies, such as avoidance, are consistently linked to increased distress and depression (Sun et al. 2020; Agha 2021). Therefore, examining both PTSD symptoms and coping responses provides a comprehensive understanding of nursing staff's psychological trajectories.

Although the psychological distress of healthcare workers has been documented, most studies have focused on cross‐sectional prevalence rather than evaluating the effectiveness of structured organizational interventions over time (Greenberg et al. 2020). There remains a lack of longitudinal evidence on whether systematic epidemic prevention programmes—which involve infection control training and resource support (Taiwan CDC 2021; WHO 2020)—can effectively mitigate PTSD symptoms and foster adaptive coping.

Addressing these issues is particularly important for nursing staff, as their psychological well‐being is closely linked to workforce stability and retention during public health crises. A better understanding of how structured prevention programmes influence these factors can provide the evidence‐based foundation needed to enhance resilience and ensure sustainable healthcare management.

To provide a theoretical foundation for the intervention, the systematic epidemic prevention programme in this study was conceptualised based on the Transactional Model of Stress and Coping (Lazarus and Folkman 1984). This theory posits that stress results from an imbalance between perceived environmental demands and available resources. Within this framework, the intervention components—such as standardised training, supply provision, and triage—aim to restructure nursing staff's primary appraisal of COVID‐19 as a manageable risk rather than a helpless threat. Concurrently, the programme strengthens their secondary appraisal by providing the necessary technical and organizational resources to cope. By enhancing these cognitive appraisal processes, the programme intends to foster adaptive coping strategies and reduce the long‐term traumatic impact of the pandemic.

## 3 The Study

### 3.1 Aims and Objectives

This study examined the effectiveness of a systematic epidemic prevention programme on the impact of events and coping response among nursing staff caring for high‐risk COVID‐19 patients using a one‐group pre‐test–post‐test design. The purposes of this study were to (1) understand the health situation among nursing staff caring for high‐risk COVID‐19 patients and to evaluate their coping responses and the impact of these events. (2) assess the effectiveness of a systematic epidemic prevention programme in improving coping responses and managing the impact of these events among the nursing staff. The systematic epidemic prevention programme includes (1) establishing a standardised care process and education training regarding the correct procedures for hand washing, PPE use, and specimen collection; (2) providing supplies (including masks, hand sanitizer, and other PPE); (3) triage and compartmentalization (grouping medical staff to fixed floors, fixed wards, fixed groups, and fixed sections, without intermixing); (4) redistributing nursing manpower (adjusting the nurse‐to‐patient ratio); (5) providing appropriate equipment (clinical carts, simple sphygmomanometer, monitors for quarantine room, and electronic thermometer) and environmental disinfection procedures (trained cleaning staff who work in fixed areas); and (6) updating the epidemic prevention plan.

## 4 Methods

### 4.1 Design

A quantitative study using a pre‐experimental study design was employed. The performance indicators included the IES‐R, GHQ, and Brief‐COPE. Symptoms associated with high‐stress situations often disappear within 6 months after the end of the high‐pressure event; therefore, the pre‐test measurements were performed on nursing staff before the intervention, and the post‐test measurements were performed 1 month, 3 months, and 6 months after the intervention.

### 4.2 Intervention

The systematic epidemic prevention programme was developed based on national and international infection control guidelines (e.g., Taiwan CDC, WHO) and previous empirical evidence from epidemic response studies. The programme was further refined through discussions with clinical experts, including senior nursing staff, infection control specialists, and hospital administrators, to ensure its relevance and applicability in real‐world clinical settings. The intervention spanned a total of 6 months. One month prior to the implementation, all participants underwent intensive education and training to ensure each individual had a thorough and accurate command of the programme content. Evaluation was conducted through multiple time points: a pre‐test was administered before the intervention, followed by post‐tests at 1 month, 3 months, and 6 months after the initiation of the programme (Table 1). Prior to formal implementation, the feasibility of the programme was evaluated through pilot discussions and administrative review, focusing on practicality, resource availability, and staff compliance. Minor adjustments were made based on feedback to enhance clarity and feasibility in clinical practise.

**TABLE 1:** Study timeline and schedule of measurements.

Time point	T0 (Baseline)	T1 (1 month)	T2 (3 months)	T3 (6 months)
Intervention	Ongoing intervention			
Measurements				
Personal information	✓			
IES‐R	✓	✓	✓	✓
GHQ	✓	✓	✓	✓
Brief‐COPE	✓	✓	✓	✓

The systematic epidemic prevention programme included the following component: (1) Standardised care and training: The research team and infection control specialists established standardised nursing protocols covering hand hygiene, personal protective equipment (PPE) use, and specimen collection. The intervention was delivered by the research team and infection control specialists. The research team had backgrounds in nursing research and clinical practise, while infection control specialists provided expertise in infection prevention and control. All intervention providers received standardised COVID‐19 infection prevention training prior to study initiation to ensure consistency. To ensure intervention fidelity, all study participants were required to complete standardised education and training (including physical lectures and hands‐on demonstrations) and pass a competency assessment prior to implementation. The execution of these procedures was monitored through regular assessments and observations to ensure ongoing compliance. (2) Supply provision: Essential supplies, including masks, hand sanitizers, and other PPE, were distributed and replenished regularly according to clinical needs to ensure that all participants had adequate resources. (3) Triage and compartmentalization: The research team, nursing department, and administrative units collaboratively planned and implemented a fixed personnel assignment principle (assigning staff to fixed floors, wards, groups, and care sections) to restrict cross‐area movement and reduce the risk of cross‐infection. (4) Redistribution of nursing manpower: Nurse‐to‐patient ratios were adjusted based on patient needs and workload to optimise nursing care. (5) Provision of equipment and cleaning: Necessary equipment such as clinical carts, sphygmomanometers, monitors for quarantine rooms, and electronic thermometers were provided. Additionally, trained cleaning personnel were assigned to specific areas for environmental disinfection, which was performed at scheduled intervals and documented in accordance with established protocols. (6) Plan updates: The epidemic prevention plan was regularly reviewed and updated based on new guidelines and feedback to ensure its ongoing effectiveness.

To ensure intervention fidelity and adherence, the implementation of all measures was monitored through on‐site observations, standardised checklists, and regular audits conducted by the research team and infection control specialists. Staff compliance with standardised procedures was recorded and analyzed to identify trends over time. Feedback was regularly provided to participating units to support continuous improvement. Intervention fidelity was maintained by ensuring that all components of the COVID‐19 infection prevention programme were implemented according to the study protocol. The intervention was delivered consistently to all participants without modification, ensuring consistency and integrity across units.

### 4.3 Instruments

#### 4.3.1 Impact of Event Scale‐Revised (IES‐R)

This study used the Chinese version of the IES‐R, which was introduced by Horowitz et al. (1979) to assess PTSD and translated into Chinese by Chang et al. (2014). The IES‐R is a revision of the IES (Impact of Event Scale), which expanded from 15 questions to 22 questions and added an assessment of symptoms related to hyperarousal. The IES‐R can measure the three major symptoms of PTSD better than the IES, including the re‐experience of trauma (invasion), emotional numbness (avoidance), and hypervigilance (hyperarousal). This study obtained approval from Dr. Tsai to use the Chinese version of the scale. The IES‐R uses a 5‐point Likert scale for each question, ranging from 0 (absolutely not) to 4 (extremely severe). Research has found that this scale has good reliability, validity, and consistency. This scale is primarily used to evaluate the severity of PTSD symptoms, with a higher score indicating more severe PTSD. The reliability (Cronbach's α) of the IES‐R in previous studies and the current study ranges 0.87–0.92 and 0.88–0.91, respectively.

#### 4.3.2 General Health Questionnaire (GHQ)

This study used the GHQ, introduced by Goldberg and Hillier (1979) and translated into Chinese by Chang (1987). The GHQ aims to evaluate a person's physical and mental health for the past month across aspects of an unhealthy body and mind. The GHQ is comprised of 28 questions in total, and we obtained approval from Professor Chang for the use of the Chinese version. The GHQ uses a 5‐point Likert scale for each question, ranging from 1 (absolutely not) to 5 (always), for a total possible score of 140. Higher scores indicate worse mental and physical health. The Chinese version of the GHQ has demonstrated strong psychometric properties. Reliability studies have shown a Cronbach's alpha ranging from 0.80 to 0.90, and validity assessments confirm its effectiveness in evaluating general mental and physical health. The GHQ has been widely used in various Chinese populations with consistent results.

#### 4.3.3 Brief Coping Orientations to Problems Experienced (Brief‐COPE)

This study used the Brief‐COPE, introduced by Carver et al. (1989) and translated into Chinese by Jeng in 2012. We obtained approval to use the Chinese version from Jeng. The full version of the COPE contains 60 questions. The Brief‐COPE reduces this number from 60 questions to 28 questions. The Brief‐COPE assesses three domains, including approach coping (10 questions), emotional coping (10 questions), and avoidance coping (8 questions). (1) Approach coping includes active coping, positive reframing, acceptance, planning, and humour. (2) Emotional coping includes emotional support, instrumental support, venting, religion, and self‐distraction. (3) Avoidance coping includes behaviour disengagement, denial, self‐blame, and substance abuse. The Brief‐COPE uses a 4‐point Likert scale for each question, ranging from 1 (absolutely not) to 4 (always). Higher scores indicate that a given strategy is used more frequently. The Brief‐COPE, translated into Chinese, has shown good validity and reliability in Chinese populations. The reliability (Cronbach's α) of the Brief‐COPE in previous studies and the current study ranges from 0.87 to 0.89 and 0.85 to 0.89, respectively.

The Brief‐COPE was used in this study to categorise coping strategies into three domains: approach coping, emotional coping, and avoidance coping. Instead of using the original 14‐factor structure, this three‐domain categorisation was adopted based on the exploratory factor analysis results of the Chinese version (Jeng 2012), which demonstrated superior construct validity in the Taiwanese population. (1) Approach coping includes strategies like active coping and positive reframing, which are generally associated with lower anxiety (Carver et al. 1989; Glanz et al. 2015). (2) Emotional coping involves strategies such as emotional support and venting. This domain is positively correlated with anxiety, suggesting that greater use may lead to higher anxiety levels (Carver et al. 1989). (3) Avoidance coping includes strategies like denial and self‐blame, which are linked to increased anxiety (Carver et al. 1989). Detailed scoring procedures for all psychometric instruments are provided in Table S1, including scale ranges, scoring methods (total vs. mean), subscales, and cut‐off values based on prior literature.

### 4.4 Sampling and Recruitment

For data collection, a purposive sample was used. The participants were determined to be eligible by applying the inclusion and exclusion criteria to the sample population, and all participants signed informed consent. The inclusion criteria were as follows: (1) nursing staff who cared for suspected or confirmed high‐risk COVID‐19 patients, as diagnosed by specialty physicians; (2) willing to respond to the questionnaire; and (3) male or female 20 years or older. The exclusion criteria were as follows: (1) inability to complete the questionnaire, or (2) unwilling to respond to the questionnaire sample. A purposive sample was used. This study chose nursing staff members from two teaching hospitals who had experiences in caring for high‐risk COVID‐19 patients. The study was conducted at two teaching hospitals, selected due to their specialised experience and resources related to COVID‐19 care. The choice of these hospitals was based on their significant involvement in managing high‐risk COVID‐19 patients and the availability of nursing staff who had experience in caring for such patients. This setting provided an ideal environment for collecting high‐quality data relevant to our research questions and ensured that the findings would be applicable to similar clinical settings.

For this study, we estimated the sample size using G*Power software. Based on the expected effect size (0.25, considered a medium effect size), a significance level (*α* = 0.05), and statistical power (1 − *β* = 0.8), our repeated measures design required 48 participants. To account for an anticipated 20% attrition rate, the initial target sample size was estimated at 58 participants. However, during the study period, all nurses who were providing care for patients with COVID‐19 in the participating institution (*N* = 84) were screened for eligibility. As the study included all eligible nurses within the study setting, the sample can be considered a census of the accessible population. Therefore, all 84 eligible nurses were invited to participate, and this sample size was approved by the institutional review board (approval number redacted) and met ethical requirements.

### 4.5 Data Analysis

Data were collected from nursing staff identified as eligible for study inclusion who agreed to respond to the questionnaires. The entire process was anonymous. The average time required to respond to the questionnaire was 30–40 min for each person. The software SPSS 19.0 was used for data analysis. The following statistical analysis methods were applied. The questionnaire was returned anonymously in sealed envelopes: (1) Descriptive and frequency statistics were used to analyse the distribution of variables, including demographic information (the scores of the IES‐R, the GHQ, and the Brief‐COPE.); (2) Generalised estimating equations (GEE) were used to evaluate the effects of the intervention after controlling for covariates (e.g., age and seniority). To specify the statistical approach, we employed a linear link function with a normal distribution for all continuous outcomes. An exchangeable working correlation structure was used to account for the dependencies among repeated measurements. In this analysis, post‐traumatic stress symptoms (IES‐R) and coping responses (Brief‐COPE) were pre‐specified as primary outcomes, while general psychological distress (GHQ) was defined as a secondary outcome. To control for the inflation of Type I error due to multiple comparisons across the four time points (M0, M1, M3, and M6), Bonferroni corrections were applied to all post hoc pairwise comparisons. The GEE results are presented as beta values (ß), 95% confidence intervals (CIs), and *p* values; (3) Pearson's correlation coefficient was used to examine correlations between demographic information and outcome variables, including impact of event, physical and mental health, and coping strategies; (4) Independent samples *t*‐test was used to examine the differences in demographic information and outcome variables between groups (nursing ladder, marital status, child status, and education level in Table 2), including impact of events, physical and mental health, and coping strategies; (5) One‐way analysis of variance (ANOVA) was used to examine the differences among three or more groups of demographic characteristics and outcome variables (Table 2), including impact of event, physical and mental health, and coping strategies. Standardised effect sizes (Cohen's d, correlation coefficient r, and eta squared [*η* ^2^]) were calculated to complement the interpretation of statistical findings. For secondary analyses involving multiple comparisons (correlation, *t*‐test, and ANOVA), false discovery rate (FDR) correction was applied using the Benjamini–Hochberg procedure. The primary analyses were conducted using GEEs to examine changes over time. No subgroup or restricted analyses were pre‐specified, and any additional analyses were considered exploratory.

**TABLE 2:** Number, percentage, mean, and standard deviation of nursing staff caring for high‐risk COVID‐19 patients (*N* = 80).

Variable	Number	Percentage	Mean	SD
Age (y)			33	8.43
20–29	38	47.5		
30–39	26	32.5		
≥ 40	16	20.0		
Seniority (y)			10.8	8.7
Sex				
Female	73	91.2		
Male	7	8.8		
Living status				
Hospital dormitory	24	30.0		
Live with family	44	55.0		
Live in a rental apartment	12	15.0		
Nursing ladder				
≤ N2*	41	51.2		
> N2	39	48.8		
Marital status				
Single	54	67.5		
Married	26	32.5		
Dependent children				
No	51	63.8		
Yes	29	36.2		
Education level				
Associate degree	22	27.5		
Bachelor's or master's	58	72.5		
Training courses (before intervention)				
No	26	32.5		
Yes	54	67.5		
Protection training (before intervention)				
No	25	31.2		
Yes	55	68.8		

Abbreviation: SD, standard deviation.

* Level 2 of registered nurse.

### 4.6 Ethical Considerations

This study was approved by an Institutional Review Board (IRB number redacted). During the recruitment process, the researchers provided detailed explanations of the study objectives, procedures, potential risks, and benefits to ensure that participants fully understood the study. Written informed consent was then voluntarily obtained from all participants prior to data collection. To protect participants' privacy and ensure data security, all collected data were de‐identified, coded, and stored in a password‐protected secure system accessible only to the research team. Participants were informed of their right to withdraw from the study at any time without any negative consequences to their rights, interests, or the care they receive.

## 5 Results

### 5.1 Study Overview and Participant Characteristics

**FIGURE 1:**
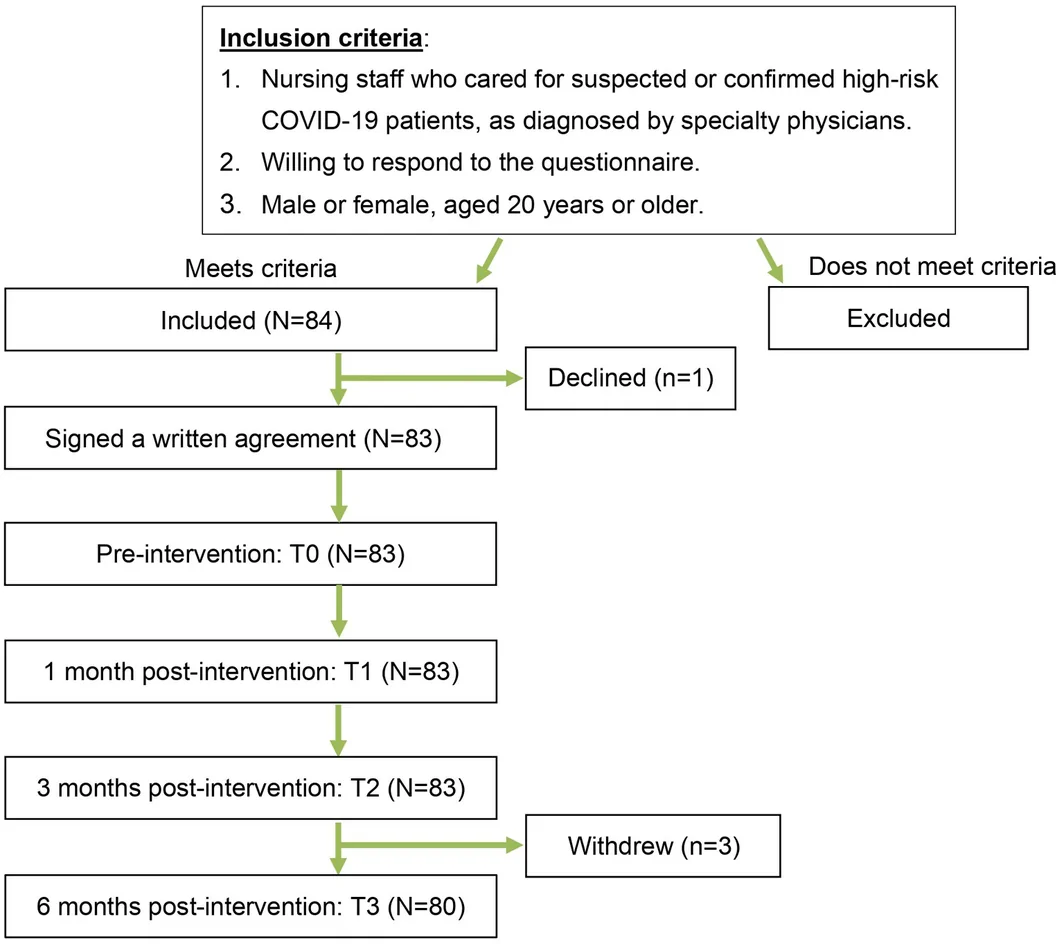
Participant flow in the one‐group pre‐test–post‐test study.

This study aimed to explore the effectiveness of a systematic epidemic prevention programme that involved nursing staff caring for high‐risk COVID‐19 patients. The interviewees were chosen from a teaching hospital in north Taiwan. The recruitment period occurred from January 2020 to September 2020, and the systematic epidemic prevention programme was implemented in February 2020. A total of 84 subjects were recruited for this study, which included 1 who was transferred to other hospitals and 3 who resigned from the hospital during the study period. The final analysis included 80 valid subjects (Figure 1), with a resultant withdrawal rate of 4.8% (4/84), indicating that the actual attrition was lower than expected and ensuring the reliability and validity of the research findings.

### 5.2 Demographic and Descriptive Statistics

Descriptive and frequency statistics were used to analyse the demographic information of participants (Table 2). The average age of the subjects was 33 years (standard deviation [SD] = 8.43 years), and 47.7% were 20–29 years old. Among the subjects, 73 were women, and 7 were men. The average nursing seniority was 10.8 years (SD = 8.7). Among the subjects, 55% lived with their family, 30% lived in hospital dormitories, and 15% lived in a rental apartment. The Clinical Ladder System ranks nurses on a scale from N0–N4 level (from level 0 to level 4 of the Nursing Clinical Ladder System. Each level has different promotion criteria, and must pass the review before being promoted, and the higher level, indicated the better ability and more responsibilities) in Taiwan. Of the study group, 51.2% of participants ranked N0–N2, and 48.8% ranked above N2. Furthermore, 67.5% of the participants were single, 32.5% were married, and 36.2% had children. Additionally, 72.5% of participants had a bachelor's or master's degree, and 27.5% had an associate degree. Among the nursing staff, 67.5% participated in training courses regarding how to care for high‐risk COVID‐19 patients before the intervention, and 68.8% participated in PPE training regarding the care for high‐risk COVID‐19 patients before the intervention. The mean number of nursing staff caring for suspected COVID‐19 patients before the intervention was 2.34 (SD = 3.23); 1 month after the intervention, the number was 5.57 (SD = 6.40); 3 months after the intervention, the number was 7.67 (SD = 9.60); and 6 months after the intervention, the number was 13.9 (SD = 19.9). Baseline characteristics were compared between participants who completed the study and those who dropped out (Table 3). No significant differences were observed in demographic information and scores between the two groups.

**TABLE 3:** Baseline characteristics of nursing staff caring for high‐risk COVID‐19 patients.

Variable	Completed (*N* = 80)		Dropout (*N* = 3)		*p*
Age (y) (mean ± SD)	32.95	8.43	36.33	12.74	0.500
20–29 (%)	38	47.5	1	33.3	
30–39 (%)	26	32.5	1	33.3	
≥ 40 (%)	16	20.0	1	33.3	
Seniority (y) (mean ± SD)	10.8	8.7	13	12.17	0.348
Sex (%)					
Female	73	91.2	3	100.0	1.000
Male	7	8.8	0	0.0	
Living status (%)					
Hospital dormitory	24	30.0	1	33.3	0.644
Live with family	44	55.0	1	33.3	
Live in a rental apartment	12	15.0	1	33.3	
Nursing ladder (%)					
≤ N2*	41	51.2	1	33.3	1.000
> N2	39	48.8	2	66.6	
Marital status (%)					
Single	54	67.5	1	33.3	0.262
Married	26	32.5	2	66.6	
Dependent children (%)					
No	51	63.8	1	33.3	0.553
Yes	29	36.2	2	66.6	
Education level (%)					
Associate degree	22	27.5	2	66.6	0.557
Bachelor's or master's	58	72.5	1	33.3	
IES‐R (mean ± SD)	0.36	0.03	0.19	0.19	0.815
GHQ (mean ± SD)	0.60	0.09	1.62	0.28	0.845
Brief‐COPE (mean ± SD)	2.30	0.44	2.35	0.36	0.867
I Approach coping	2.75	0.05	2.28	0.08	0.942
II Emotional coping	2.44	0.06	2.48	0.30	0.824
III Avoidance coping	1.55	0.02	3.36	1.26	0.408

### 5.3 Pre‐ and Post‐Intervention Scores

Descriptive statistics revealed a decrease in IES‐R and GHQ scores post‐intervention. The mean IES‐R score decreased from 0.39 (SD = 0.1) pre‐intervention to 0.32 (SD = 0.12) at 3 months, indicating reduced PTSD symptoms. Similarly, GHQ scores decreased from a mean of 1.68 (SD = 0.25) pre‐intervention to 1.48 (SD = 0.2) at 3 months, indicating reduced psychological distress.

In addition to mean differences, we examined the proportion of participants exceeding the clinically relevant threshold for psychological distress. The proportion above the GHQ cut‐off decreased from 100.0% (83/83) at baseline to 96.4% (80/83) at 3 months post‐intervention, suggesting a reduction in clinically relevant psychological distress.

The Brief‐COPE analysis indicated that approach coping remained the most frequently used strategy before and after the intervention (pre‐test *M* = 2.75, SD = 0.05; post‐test *M* = 2.75, SD = 0.05), while avoidance coping was least used (pre‐test *M* = 1.55, SD = 0.02; post‐test *M* = 1.55, SD = 0.02) (Table 4). These changes suggested small‐to‐moderate effect sizes, indicating improvements in psychological outcomes.

**TABLE 4:** The mean (*M*) and standard deviation (SD) of the IES‐R, GHQ, and Brief‐COPE at pre‐test and post‐test time points.

Variables	IES‐R		GHQ		Brief‐COPE					
					I Approach coping		II Emotional coping		III Avoidance coping	
	*M*	SD	*M*	SD	*M*	SD	*M*	SD	*M*	SD
M0	0.39	0.10	1.68	0.25	2.78	0.30	2.42	0.35	1.53	0.29
M1	0.37	0.12	1.58	0.21	2.75	0.26	2.46	0.35	1.58	0.27
M3	0.32	0.12	1.48	0.20	2.78	0.27	2.50	0.36	1.55	0.23
M6	0.37	0.10	1.65	0.22	2.67	0.28	2.36	0.29	1.55	0.25
Mean	0.36	0.03	0.60	0.09	2.75	0.05	2.44	0.06	1.55	0.02

Abbreviations: M0: Before intervention; M: mean; SD: standard deviation; IES‐R, Impact of Event Scale‐Revised; GHQ, General Health Questionnaire; Brief‐COPE: Brief Coping Orientations to Problems Experienced; M1: 1 month after the intervention; M3: 3 months after the intervention; M6: 6 months after the intervention.

### 5.4 Effectiveness of the Systematic Epidemic Prevention Programme

Regarding the intervention outcomes, IES‐R scores at 3 months post‐intervention showed a reduction compared with baseline; however, the change was not statistically significant (*β* = −1.54, *p* = 0.23), suggesting a non‐significant trend with a small effect size. GHQ scores showed statistically significant reductions at 1 month and 3 months post‐intervention (*β* = −2.89, *p* = 0.02; *β* = −5.45, *p* = 0.001), indicating moderate effect sizes and clinically relevant improvements in general psychological distress. These findings correspond to moderate effect sizes and indicate improvements in overall mental and physical health. In addition, emotional coping techniques increased significantly at 3 months post‐intervention (*β* = 0.83, *p* = 0.04), reflecting a small effect size (Table 5).

**TABLE 5:** GEE analysis of the effects of the intervention on the IES‐R, GHQ, and Brief‐COPE scores comparing pre‐test and post‐test values.

Item	M1–M0			M3–M0			M6–M0		
	*β*	95% CI	*p*	*β*	95% CI	*p*	*β*	95% CI	*p*
IES‐R	−0.34	[−2.05, 1.35]	0.70	−1.54	[−4.05, 0.99]	0.23	−0.45	[−3.07, 2.17]	0.74
GHQ	−2.89	[−5.33, −0.56]	0.02*	−5.45	[−8.61, −2.36]	0.001**	−0.89	[−5.27, 3.50]	−0.45
Brief COPE	0.45	[1.91, −1.01]	0.54	0.95	[−0.81, 2.71]	0.29	−1.55	[−5.92, 2.82]	0.49
I Approach Coping	−0.40		0.32	−0.07		0.87	−0.93		0.38
II Emotional Coping	0.41		0.26	0.83		0.04*	−0.63		0.51
III Avoidance Coping	0.44		0.16	0.27		0.46	0.25		0.60

Abbreviations: M0: Before intervention; IES‐R, Impact of Event Scale‐Revised; GHQ, General Health Questionnaire; Brief‐COPE: Brief Coping Orientations to Problems Experienced; M1: 1 month after the intervention; M3: 3 months after the intervention; M6: 6 months after the intervention.

* *p* < 0.05.

** *p* < 0.01.

### 5.5 Box Plot Analysis

Box plots were used to present the distribution of GHQ scores and emotional coping scores at all four time points before and after the intervention. For the GHQ, post‐test scores were lower than the pre‐test scores, with significant decreases observed at 1 month and 3 months after the intervention. Based on Brief‐COPE scores, emotional coping scores increased significantly at 3 months post‐intervention.

**FIGURE 2:**
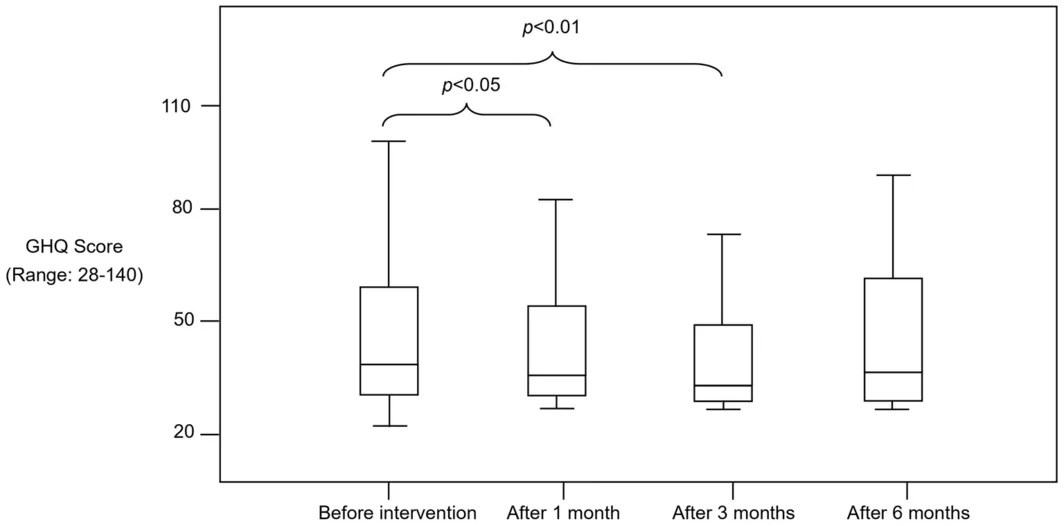
Box plot of the GHQ scores at pre‐test and post‐test time points.

Figure 2 (score range: 28–140) shows that the minimum and median GHQ scores were approximately the same at all four time points. However, the maximum scores and the range of scores decreased 1 month and 3 months after the intervention, indicating reduced psychological distress.

**FIGURE 3:**
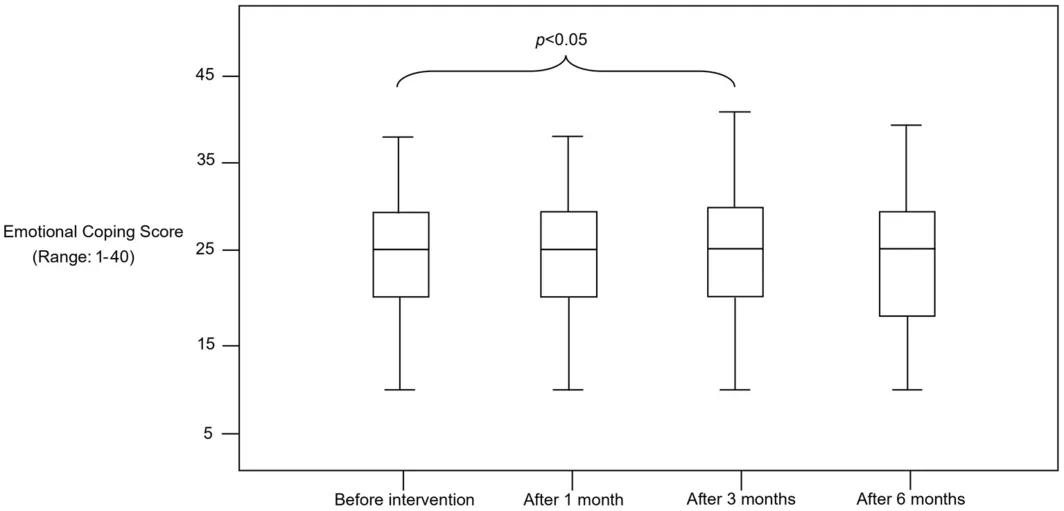
Box plot of the emotional coping scores at pre‐test and post‐test time points.

In addition, Figure 3 (score range: 1–40) shows variability in emotional coping scores across time points, with increased dispersion observed at 3 months post‐intervention, suggesting greater heterogeneity in coping responses.

### 5.6 Correlations and ANOVA Analysis

Given the multiple comparisons performed in these secondary analyses, FDR correction was applied to control for type I error. Some associations did not remain statistically significant after correction and should therefore be interpreted as exploratory. Significant differences in GHQ and emotional coping scores were observed at 1 and 3 months after the systematic programme intervention. Therefore, independent samples *t*‐tests, Pearson's correlation, and ANOVA were conducted to examine the relationships between demographic characteristics, physical and mental health, and coping strategies. The results showed no significant differences according to sex, nursing rank, marital status, education level, or having children. GHQ scores at 1 month post‐intervention were negatively correlated with age (*r* = −0.22, *p* = 0.04), and GHQ scores at 3 months post‐intervention were negatively correlated with age (*r* = −0.30, *p* = 0.008) and seniority (*r* = −0.29, *p* = 0.008; Table 6). These correlations (*r* = −0.22 to −0.30) indicate small‐to‐moderate effect sizes and suggest that as age and seniority increased, GHQ scores decreased, indicating lower levels of mental distress among more experienced nursing staff. However, these findings should be interpreted with caution.

**TABLE 6:** Pearson's correlation analysis GHQ and emotional coping scores with basic information.

Variables	Age		Seniority	
	*r*	*p*	*R*	*p*
GHQ				
M1–M0	−0.22	0.04*	−0.21	0.06
M3–M0	−0.30	0.008**	−0.29	0.008**
Emotional coping				
M3–M0	−0.08	0.46	−0.11	0.35

Abbreviations: M0: Before intervention; GHQ: General Health Questionnaire; M1: 1 month after intervention; M3: 3 months after intervention; M6: 6 months after intervention.

* *p* < 0.05.

** *p* < 0.01.

The ANOVA revealed a significant difference in emotional coping strategies according to living conditions at 3 months post‐intervention (*F* = 4.96, *p* = 0.009). Post hoc analysis using the Scheffé test indicated that nursing staff living in hospital dormitories had significantly lower emotional coping scores than those living in rental apartments (*p* = 0.007; Table 7). This finding suggests that nursing staff living in rental housing may have used emotional coping strategies more frequently, possibly reflecting higher levels of anxiety. However, this result should also be interpreted with caution.

**TABLE 7:** ANOVA analysis of GHQ and emotional coping scores with demographic information.

Variables	Number	Mean	SD	*F*	Scheffe
GHQ					
M1–M0					
Hospital dormitory	24	−5.17	10.82	0.91	
Living with family	44	−1.52	11.26		
Living in a rental apartment	12	−3.67	9.14		
M3–M0					
Hospital dormitory	24	−6.79	12.48	0.41	
Living with family	44	−4.41	12.81		
Living in a rental apartment	12	−8.00	21.28		
Emotional coping					
M3–M0				4.96**	(3) > (1)
Hospital dormitory	24	−0.58	4.07		
Living with family	44	0.95	2.97		
Living in a rental apartment	12	3.17	3.35		

Abbreviations: M0: Before intervention; SD: standard deviation; GHQ: General Health Questionnaire; M1: 1 month after the intervention; M3: 3 months after the intervention; M6: 6 months after the intervention.

** *p* < 0.01.

However, these findings were derived from secondary analyses and should be interpreted with caution. For secondary analyses, FDR correction was applied using the Benjamini–Hochberg procedure, and findings that did not remain statistically significant after correction were interpreted as exploratory.

## 6 Discussion

The findings are consistent with previous evidence on psychological stress among nurses caring for patients with COVID‐19 and support the role of resilience theory in explaining improved coping and psychological outcomes. These results highlight the importance of ongoing psychological and organizational support for frontline nurses. Overall, the mean scores for the IES‐R and GHQ decreased following the intervention with the systematic epidemic prevention programme. The reduction in GHQ scores demonstrated moderate effect sizes, whereas the change in IES‐R scores was small and not statistically significant. This distinction is crucial, as PTSD symptoms (measured by IES‐R) and general psychological distress (measured by GHQ) represent different psychopathological constructs. This indicates that while the nursing staff's general mental distress was alleviated, the specific traumatic impact related to COVID‐19 care followed a different recovery trajectory. Lai et al. (2020) conducted a cross‐sectional study showing that nursing staff on the front lines in Wuhan had higher IES‐R scores compared to those in other regions, highlighting the severe mental health impact of the pandemic. Conversely, Chen et al. (2020) demonstrated that targeted interventions could significantly reduce psychological distress among healthcare workers. Our results align with these findings, showing that the IES‐R scores in our study were lower than those reported by Lai et al. (2020).

The reduction in IES‐R scores, though non‐significant, can be explained by the intervention's capacity to increase “perceived controllability” and “professional self‐efficacy.” PTSD symptoms, such as intrusion and hyperarousal, are often driven by a sense of helplessness and perceived life threat. By providing systematic epidemic prevention training and clear protective protocols, the intervention likely reduced the staff's perception of threat and restored a sense of agency, which are essential mechanisms for mitigating post‐traumatic stress (Lai et al. 2020).

Our study's observation that nursing staff utilised approach coping techniques is consistent with Huang et al. (2020), who found that more experienced and knowledgeable nursing staff employed proactive coping strategies during the COVID‐19 outbreak. This suggests that well‐designed interventions can help staff adopt effective coping mechanisms in high‐stress situations. Furthermore, Spoorthy et al. (2020) supported these findings, noting that approach coping is associated with better mental health outcomes among healthcare workers in crisis situations, although the magnitude of change was modest.

The significant decrease in GHQ scores 3 months after the intervention reflects notable improvements in both physical and mental health among the nursing staff. The GHQ primarily captures emotional suffering and functional interference, which responded more readily to the systematic support provided. The fact that all staff participated in epidemic prevention and protective training by 3 months' post‐intervention underscores the importance of comprehensive training and resources. The increased stress levels among frontline healthcare workers during the COVID‐19 pandemic were also addressed, with shared experiences highlighting various strategies for building resilience and maintaining well‐being. Key strategies included mindfulness, gratitude, self‐care, and social support (Liljestrand and Martin 2021). The observed improvements may also reflect the overall decline in COVID‐19 cases in Taiwan, as reported by the Taiwan CDC (2021), suggesting that pandemic conditions can influence psychological outcomes.

Wu et al. (2020) emphasised the importance of formulating effective epidemic prevention strategies and fostering cross‐industry collaboration in managing stress during public health emergencies. Our study supports this perspective, demonstrating enhanced coping responses and reduced mental distress as benefits of systematic epidemic prevention programmes. However, it is important to note that while general distress improved, the persistent nature of PTSD symptoms may require more specialised, trauma‐focused psychological support beyond systematic organizational training.

The observation that age and seniority were negatively correlated with physical and mental distress aligns with Liu and Liu (2009), who indicated that older and more experienced staff generally cope better with work‐related stress. These correlations were small to moderate in magnitude, suggesting a modest association between experience and psychological distress. Similarly, Chian (2005) found that increased experience and age contribute to better stress management capabilities, which is consistent with our findings.

Additionally, our study found that nursing staff living in rental apartments experienced higher anxiety levels compared to those in hospital dormitories. This finding is supported by Feng et al. (2020), who reported concerns about living conditions and the risk of virus transmission. This underscores the need for hospitals to consider staff living conditions as part of their support strategies, highlighting the importance of addressing environmental factors that may contribute to staff anxiety during the pandemic.

Where established cut‐off values were available, changes were interpreted in relation to clinical thresholds; however, minimal clinically important difference (MCID) values were not uniformly available across all instruments used in this study.

In summary, this study demonstrates that the systematic epidemic prevention programme effectively reduced the psychological impact of COVID‐19 on nursing staff by improving general mental health, partially mitigating PTSD symptoms, and enhancing coping strategies. The incorporation of recent literature supports these findings and emphasises the ongoing need for targeted interventions and support for healthcare workers during public health crises. However, these findings were derived from secondary analyses and should be interpreted with caution.

### 6.1 Limitations

Several limitations of this study should be acknowledged. First, the study used a quasi‐experimental design without a control group, which limits the ability to rule out natural recovery or other external factors as causes of the observed changes. Second, this study was conducted in a single institution; therefore, the findings may not be generalizable to all nursing staff caring for high‐risk COVID‐19 patients in other settings. Third, the relatively small sample size may have limited the statistical power to detect significant effects in certain outcomes; specifically, the changes in post‐traumatic stress symptoms (IES‐R) and overall coping responses (Brief‐COPE) did not reach statistical significance, despite the significant improvement observed in general psychological distress (GHQ). Fourth, the study was conducted during the early phase of the pandemic, and external contextual changes over time may have influenced participants' psychological status and coping responses. Finally, self‐reported questionnaires were used to measure psychological outcomes, which may be subject to response bias.

## 7 Conclusions

This study examined the effects of a systematic epidemic prevention programme on the mental health status and coping responses of nursing staff caring for high‐risk COVID‐19 patients. The intervention was associated with a reduction in post‐traumatic stress symptoms and significantly improved general psychological symptoms among nursing staff, while assisting them in managing nursing care stress. The results of this study can be used as a reference for clinical care in response to similar infectious diseases, facilitating the rapid implementation of systemic epidemic prevention programmes in the future. Furthermore, a systemic epidemic prevention programme can improve the coping abilities of nursing staff, which may also improve the quality of caring and improve the recovery of high‐risk COVID‐19 patients. Recommendations for clinical practise include the following. (1) Hospitals should conduct regular training for nursing staff caring for COVID‐19 patients, including rolling adjustment training that covers the concepts of disease, PPE use, triage, compartmentalization, and fixed manpower. (2) Hospitals should sustain established horizontal and vertical integration mechanisms for the cross‐regional reorganisation of resources during epidemic prevention and response, including nursing manpower, to facilitate the rapid redistribution of resources during preparation and contingency periods in the future. (3) Hospitals should provide professional psychological consultations to nursing staff, offering the staff opportunities to express their emotions and reduce psychological stress. The current COVID‐19 epidemic still affects people around the world. For the nursing staff who provide the first‐line care of high‐risk COVID‐19 patients, it is recommended to apply the complete systemic epidemic prevention programme in order to reduce the coping response effectively.

## Author Contributions

**Shui‐Tao Hu:** conceptualization, methodology, validation, formal analysis, investigation, data curation, writing – original draft, writing – review and editing, supervision. **Wen‐Pin Yu:** methodology, validation, formal analysis, investigation, writing – original draft, writing – review and editing. **Fang‐Kuei Hsieh:** validation, formal analysis, investigation, writing – original draft, writing – review and editing. **Chien‐Tzung Chen:** methodology, validation, formal analysis, investigation, writing – original draft, writing – review and editing.

## Funding

This study was funded by the Chang Gung Medical Research Program of Keelung, Taiwan (grant CMRPG2K0291).

## Ethics Statement

This study was approved by the institutional review board of Chang Gung Memorial Hospital, Keelung. Approval number: 202000499B0.

## Conflicts of Interest

The authors declare no conflicts of interest.

## Supporting information


**Table S1:** Scoring rules for IES‐R, GHQ‐28 and Brief‐COPE scales.

## Data Availability

The data that support the findings of this study are available from the corresponding author upon reasonable request.
