# Supplementary material for: Effectiveness of a Systematic Epidemic Prevention Program on the Coping Response of Nursing Staff Caring for High‐Risk COVID‐19 Patients
Source: Nurs Open. 2026 Jun 4;13(6):e70600. doi: 10.1002/nop2.70600 (PMC13581082; doi:10.1002/nop2.70600)
Supplement: Supplementary file 1 [file NOP2-13-e70600-s001.docx]

Table S1. Scoring rules for IES-R, GHQ-28 and Brief-COPE scales

| **Instrument** | **Scale range** | **Total scores** | **Average scores** | **Subscales** | **Cut-off points** |
| --- | --- | --- | --- | --- | --- |
| **IES-R** | 0 (absolutely not) to 4 (extremely severe) (Likert scale) | 88  (22 items) | Total sum/22 | None (Total score used for analysis) | A total score of ≥ 25 (equivalent to an average score of ≥ 1.14) indicates the presence of significant PTSD symptoms (Chang et al., 2014). |
| **GHQ** | 1 (absolutely not) to 5 (always) (Likert scale) | 140  (28 items) | Total sum/28 | None (Total score used for analysis) | No clinical cut-off was applied. Higher scores indicate worse mental and physical health. |
| **Brief-COPE** | 1 (absolutely not) to 4 (always) (Likert scale) | 112  (28 items) | Total sum/28 | 1. Approach coping  (20 items)  2. Emotional coping  (20 items)  3. Avoidance coping  (8 items) | No clinical cut-off was applied. Higher scores indicate that a given strategy is used more frequently. |
